# STAT1-induced regulation of lncRNA ZFPM2-AS1 predicts poor prognosis and contributes to hepatocellular carcinoma progression via the miR-653/GOLM1 axis

**DOI:** 10.1038/s41419-020-03300-4

**Published:** 2021-01-04

**Authors:** Xi-wu Zhang, Qiu-han Li, Zuo-di Xu, Jin-jin Dou

**Affiliations:** 1 Department of pharmacy, Heilongjiang University of Chinese Medicine, Harbin, Heilongjiang China; 2 Department of Medicine, First affiliated Hospital, Heilongjiang University of Chinese Medicine, Harbin, Heilongjiang China

**Keywords:** Cancer therapy, Oncogenes

## Abstract

Long noncoding RNAs (lncRNAs) have drawn growing attention owing to their important effects in various tumors, including hepatocellular carcinoma (HCC). Recently, a newly identified lncRNA, ZFPM2 antisense RNA 1 (ZFPM2-AS1), was reported to serve as an oncogene in gastric cancer. However, its function in tumors remains largely unknown. In this study, we identified ZFPM2-AS1 as a novel HCC-related lncRNA, which was observed to be distinctly upregulated in HCC tissues and associated with shorter overall survival. Luciferase reporter and chromatin immunoprecipitation assays suggested that overexpression of ZFPM2-AS1 was induced by STAT1. Functional investigations suggested that the inhibition of ZFPM2-AS1 suppressed cell proliferation, metastasis, cell cycle progression while accelerated cell apoptosis. Mechanistic studies showed that there were two binding sites of miR-653 within the sequence of ZFPM2-AS1 and the levels of ZFPM2-AS1 were negatively correlated with miR-653. In addition, ZFPM2-AS1 could reverse the suppressor effects of miR-653 on the proliferation and metastasis of HCC cells by the modulation of GOLM1, a target gene of miR-653. To conclude, we provided a better understanding of the interaction mechanism between ZFPM2-AS-miR-653-GOLM1 axis, which may help develop prognostic biomarkers and therapeutic target for HCC.

## Introduction

Hepatocellular carcinoma (HCC) is the sixth most common malignant tumor and third leading cause of tumor-associated death all over the world^1^. There are many studies showing the possible risk factors involved in the canceration of liver and evidences from China and Southeast Asia indicate that viral hepatitis B and cirrhosis are most common events in tumor incidence^2,3^. Although a large number of financial resources have been devoted to the studies of curative treatments targeting HCC, which has resulted in continual advancements in HCC in the past few decades, the long-term survival time of HCC patients remains relatively poor^4,5^. One of the important reasons for the poor clinical outcome is that many patients are diagnosed at an advanced stage, which was highly associated with metastasis of HCC cells^6^. Thus, further exploration of the molecular mechanisms underlying HCC development was of importance for the discovery of more effective diagnosis methods and novel anti-cancer targets.

Long noncoding RNAs (lncRNAs) are a class of RNAs molecules that are longer than 200 nucleotides in length^7^. Previous studies on the functional research of lncRNAs reveal that lncRNAs were “transcriptional noise” due to the lack of an open reading frame, which results in limited protein-coding abilities^8^. With the huge success of high-throughput sequencing, more and more dysregulated lncRNAs were identified and several classic lncRNAs have been functionally characterized^9^. The regulator effects of lncRNAs in gene expressions at several levels, such as chromatin modification, transcription, and posttranscriptional processing, suggesting their potential roles in biological progress^10,11^. In recent years, owing to the involvement of lncRNAs in the modulation of tumor-related genes, lncRNAs have been confirmed to be involved in tumor progression by regulating cell growth, apoptosi1s, and metastasis^12^. Those previous findings suggest that functional lncRNAs could be utilized for tumor diagnosis and prognosis^13^. However, a large number of lncRNAs remain to be functionally characterized.

LncRNA ZFPM2 antisense RNA 1 (ZFPM2-AS1) was a newly discovered lncRNA involved in the tumor progression. A previous study by Kong et al.^14^ first reported that ZFPM2-AS1 was highly expressed in gastric cancer and acted as a tumor promoter by promoting cell proliferation and metastasis. Then, the possible prognostic value of ZFPM2-AS1 in HCC was also reported by analyzing TCGA data sets^15^. However, the potential function and molecular mechanism in HCC remain largely unclear.

## Results

### Upregulation of ZFPM2-AS1 in HCC tissues and cell lines

**Fig. 1:**
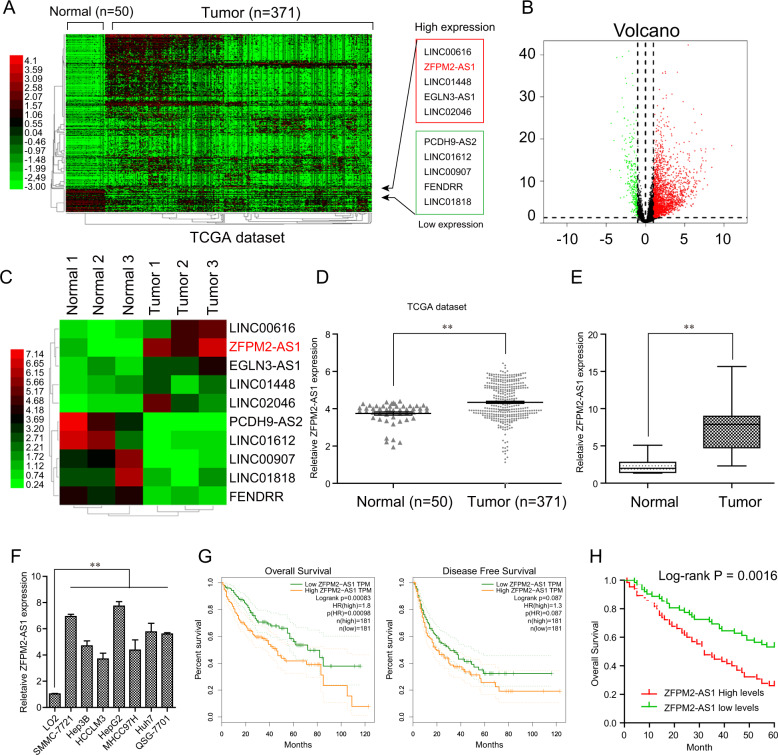
ZFPM2-AS1 was upregulated in HCC specimens and cells. **A** Heatmap of differentially expressed lncRNAs in HCC samples using TCGA data set analysis. **B** Volcano map of differentially expressed lncRNAs. **C** Real-time PCR assays detected the levels of selected five upregulated and five downregulated lncRNAs in three paired HCC tumor samples and adjacent normal tissues. The results showed by heatmap form. **D** Relative ZFPM2-AS1 expression in HCC specimens using TCGA data set analysis. **E** Real-time PCR analyses determined ZFPM2-AS1 levels in 127 paired HCC tumor specimens and adjacent normal samples. **F** ZFPM2-AS1 levels in HCC cell lines were evaluated by qPCR analyses. The L02 cell line was used as a reference. **G** GEPIA algorithm analyzed the overall survival and disease-free survival of HCC patients with high or low expression of ZFPM2-AS1. **H** Overall survival analyses of 127 HCC patients with high or low ZFPM2-AS1 expression. Data are presented as the mean ± SD from three independent experiments. *P < 0.05, **P < 0.01.

For the identification of dysregulated lncRNAs in HCC tissues, we downloaded microarray data from TCGA data sets and performed “R studio” software for statistical analysis. We found that many abnormal expressed lncRNAs in HCC shown using HeatMap and volcano plot and ZFPM2-AS1 was one of the most upregulated lncRNAs (Fig. 1A and B). Then, we chose several dysregulated lncRNAs for the examination of their levels in three normal samples and HCC samples using RT-PCR. As expected, ZFPM2-AS1 expression was highly expressed in HCC tissues (Fig. 1C). In addition, the data of TCGA also supported the upregulation of ZFPM2-AS1 in HCC tissues (Fig. 1D). To further confirm the above results, we further performed RT-PCR, finding that ZFPM2-AS1 levels in healthy liver were significantly lower than that in the HCC tissues (Fig. 1E, *p* < 0.01). In addition, we also observed that the expression of ZFPM2-AS1 was distinctly upregulated in all seven human HCC cell lines compared with the L02 cell line (Fig. 1F).

### Prognostic values of ZFPM2-AS1 expression in HCC

To explore the clinical significance of ZFPM2-AS1 in HCC, we divided 127 patients from our hospital into two groups (high, *n* = 64 and low, *n* = 63) based on the mean levels of ZFPM2-AS1 in all HCC samples. The results indicated that a high expression of ZFPM2-AS1 was significantly correlated with vein invasion (*p* = 0.010) and TNM stage (*p* = 0.026) (Table 1). Then, we further explored the possible roles of ZFPM2-AS1 on the overall survival of HCC patients. Using an online software “GEPIA”, which can be used to analyze the survival data from TCGA data sets. As shown in Fig. 1G, we found that patients with higher levels of ZFPM2-AS1 displayed shorter 5-year overall survival. On the other hand, high expressions of ZFPM2-AS1 were correlated with shorter overall survival of patients (*p* = 0.0016, Fig. 1H). Moreover, the result of univariate assays revealed that ZFPM2-AS1 expression, vein invasion, and TNM stage were distinctly associated with a shorter survival rate in patients with HCC (*p* < 0.05). More importantly, the multivariate analysis confirmed that increased ZFPM2-AS1 expression was an independent unfavorable prognostic factor for HCC patients (HR = 2.834, 95% CI: 1.271–4.278, *p* = 0.013, Table 2).

**Table 1:** The association between ZFPM2-AS1 expression and characteristics of patients suffering from HCC.

Clinicopathological features	Number of cases	ZFPM2-AS1 expression		*p* value
		High	Low	
*Gender*				0.330
Male	61	28	33	
Female	66	36	30	
*Age*				0.799
<50	74	38	36	
≥50	53	27	27	
*Tumor size (cm)*				0.218
<5	82	38	44	
≥5	45	26	19	
*Serum AFP (μg/L)*				0.330
<400	53	24	29	
≥400	74	40	34	
*Tumor differentiation*				0.094
Well+moderate	88	40	48	
Poor	39	24	15	
*Tumor number*				0.167
Single	77	35	42	
Multiple	50	29	21	
*Vein invasion*				0.010
No	94	41	53	
Yes	33	23	10	
*TNM stage*				0.026
I–II	87	38	49	
III–V	40	26	14

**Table 2:** Univariate and multivariate analysis of overall survival in HCC patients.

Variables	Univariate analysis			Multivariate analysis		
	HR	95% CI	*p*	HR	95% CI	*p*
Gender	1.562	0.774–2.128	0.352	—	—	—
Age	1.284	0.572–1.982	0.218	—	—	
Tumor size	1.486	0.893–2.582	0.137	—	—	—
Serum AFP	1.583	0.635–2.382	0.119	—	—	—
Tumor differentiation	1.428	0.774–2.328	0.189	—	—	—
Tumor number	0.895	0.569–1.885	0.128	—	—	—
Vein invasion	3.263	1.366–4.731	0.009	2.884	1.184–4.364	0.015
TNM stage	3.372	1.428–4.466	0.013	2.742	1.134–4.018	0.018
ZFPM2-AS1 expression	3.127	1.492–4.778	0.011	2.834	1.271–4.278	0.013

### STAT1 bound to the promoter region of ZFPM2-AS1 and upregulated its expression in HCC cells

**Fig. 2:**
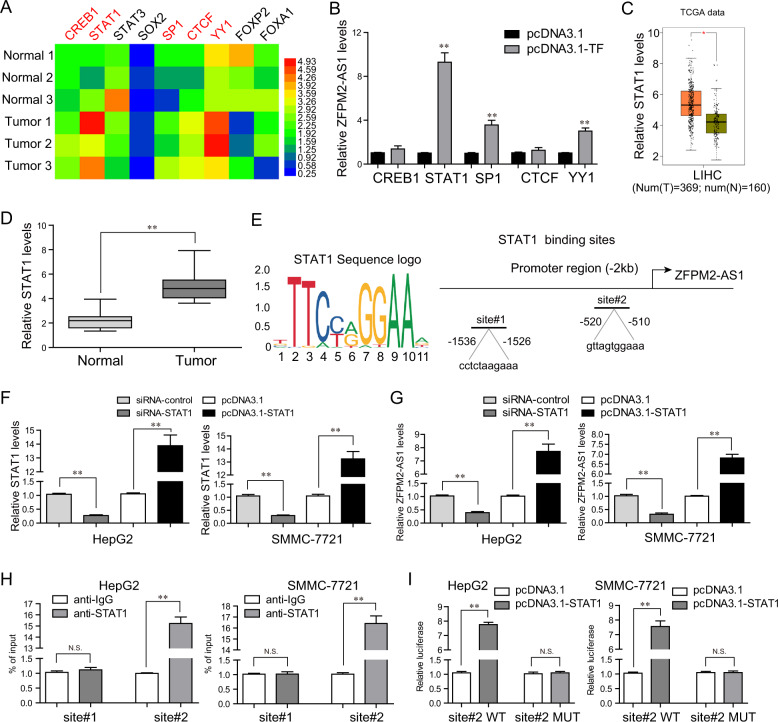
STAT1 activated ZFPM2-AS1 upregulation in HCC. **A** Real-time PCR assays evaluated the expression of a panel of transcriptional factors in three paired HCC tumor samples and normal tissues. The results were presented using heatmap form. **B** Real-time PCR assays determined the levels of ZFPM2-AS1 in HepG2 cells after, respectively, overexpressing CREB1, STAT1, SP1, CTCF, and YY1. **C** Relative STAT1 expression in HCC was assessed by GEPIA algorithm using TCGA data. **D** Relative STAT1 levels in HCC specimens from 127 patients. **E** JASPAR algorithm predicted two biding sites (site#1, site#2) between STAT1 and ZFPM2-AS1 promoter regions. **F** Relative STAT1 levels in HepG2 and SMMC-7721 cells were detected by qPCR analyses after various treatment. **G** Real-time PCR assays examined ZFPM2-AS1 expression in HepG2 and SMMC-7721 cells after STAT1 was overexpressed or knocked down. **H** ChIP assays. **I** Luciferase reporter assays detected the luciferase activities in HCC cells after transfection with site#2 wild-type (WT) reporter plasmids or site#2 mutant-type (MUT) reporter plasmids. Data are presented as the mean ± SD from three independent experiments. *P < 0.05, **P < 0.01.

We next sought to uncover the molecular mechanisms, which contributed to the upregulation of ZFPM2-AS1 in HCC. Recently, accumulating evidence revealed that some transcriptional factors (TFs) might be involved in modulating lncRNAs transcription via directly binding to their promoter regions^16^. As the data presented in Fig. 2A, mRNA levels of CREB1, STAT1, SP1, CTCF, and YY1 were remarkably upregulated in HCC tumor samples. Afterward, we conducted qPCR analyses and the data demonstrated that only STAT1, SP1, and YY1 were able to induce ZFPM2-AS1 expression and STAT1 could enhance the expression of ZFPM2-AS1 at the highest levels (Fig. 2B). Therefore, we next aimed to investigate whether STAT1 was the exact stimulator of ZFPM2-AS1 expression in HCC cells. In fact, both bioinformatics analyses using TCGA data and qPCR analyses using 127 paired HCC tissue samples confirmed that STAT1 was significantly upregulated in HCC tumor specimens (Fig. 2C and D). Then, we employed “JASPAR” algorithm (http://jaspar.genereg.net/) to predict the binding sites of STAT1 in ZFPM2-AS1 promoter regions, and we selected two possible binding sites (site#1, site#2) for further study (Fig. 2E). We next detected the STAT1 expression in HCC cells after transfecting STAT1 siRNAs (siRNA-STAT1) or pcDNA3.1-STAT1 (Fig. 2F). Subsequently, the results of qPCR suggested that enhancing expression of STAT1 was able to increase ZFPM2-AS1 expression, whereas STAT1 knockdown could lead to remarkable decline of ZFPM2-AS1 levels, which indicated that STAT1 was capable to stimulate the expression of ZFPM2-AS1 in HCC cells (Fig. 2G). Then, the results of chromatin immunoprecipitation (ChIP) assays confirmed that STAT1 physically bound to the promoter region of ZFPM2-AS1 in HCC cells and we observed marked enrichment of site#2 by STAT1 antibody when compared with the negative control (Fig. 2H). After, respectively, transfecting the luciferase reporter plasmids into HCC cells, increased STAT1-binding activity on the site#2 region of ZFPM2-AS1 promoter was observed by luciferase reporter assays (Fig. 2I). In conclusion, our findings proved that STAT1 activated the transcription of ZFPM2-AS1 in HCC.

### ZFPM2-AS1 depletion depressed malignancy phenotypes of HCC cells in vitro

**Fig. 3:**
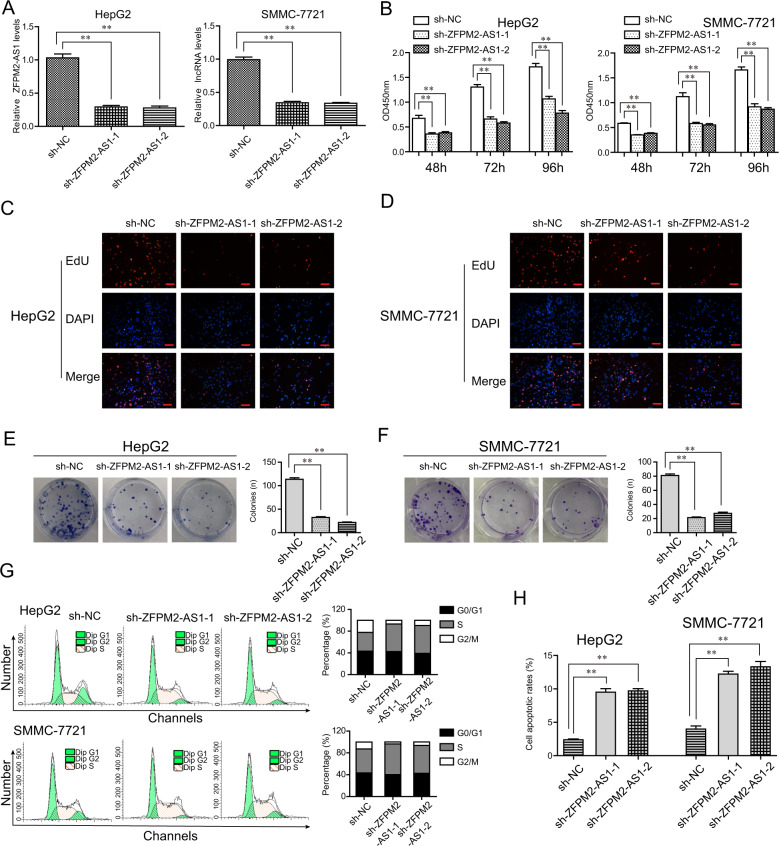
ZFPM2-AS1 depletion suppressed cell growth and promoted cell apoptosis in HCC. **A** HepG2 and SMMC-7721 cells were infected with shRNA lentivirus specifically targeting ZFPM2-AS1 (sh-ZFPM2-AS1-1, sh-ZFPM2-AS1-2), and the shRNA-knockdown efficiency was evaluated by qPCR analyses. **B** The proliferation of HCC cells after infection was determined by CCK-8 assays. **C**, **D** EdU assays examined the proliferative cells (red). DAPI was used to stain the cellular nuclei (blue). Scale bar = 200 μm. **E**, **F** Colony formation assays. **G** Flow cytometry analyzed the cell cycle of HCC cells after infection. **H** Cell apoptosis was determined by flow cytometry. Data are presented as the mean ± SD from three independent experiments. *P < 0.05, **P < 0.01.

Next, we sought to explore the impact of ZFPM2-AS1 on the malignancy development of HCC cells. The data from qPCR analyses indicated that ZFPM2-AS1 shRNA plasmids transfection led to ZFPM2-AS1 knockdown with an efficiency of >65% in HCC cells (Fig. 3A). Subsequently, the results of CCK-8 assays showed that measurement of OD 450 nm absorbance values validated that the proliferation rates of ZFPM2-AS1 depleted cells presented a notable decrease relative to that of the control cells (Fig. 3B). The results of EdU staining also confirmed the proliferation results. The data proved that repressing ZFPM2-AS1 levels markedly reduced the number of proliferative HCC cells (Fig. 3C and D). Moreover, clonogenic assays demonstrated that the clone formation abilities were also attenuated upon ZFPM2-AS1 depletion (Fig. 3E and F). Hence, to discover the antiproliferation mechanisms of ZFPM2-AS1 knockdown, flow cytometry analyses were conducted and the results presented that ZFPM2-AS1 knockdown resulted in a cell cycle arrest at S-phase (Fig. 3G). Moreover, the percentages of apoptotic cells were significantly increased in HCC cells after ZFPM2-AS1 deficiency (Fig. 3H).

### ZFPM2-AS1 knockdown suppressed the growth of HCC tumors in vivo

**Fig. 4:**
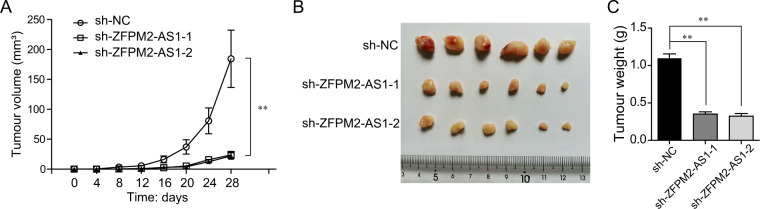
The impact of ZFPM2-AS1 on HCC tumorigenesis in vivo. **A** Effects of ZFPM2-AS1 depletion in HepG2 cells on subcutaneous tumor growth. Tumor volumes were measured every 4 days. **B** Images of whole tumors from the nude mice injected with HepG2 cells stably infected with sh-NC, sh-ZFPM2-AS1-1, or sh-ZFPM2-AS1-2 lentivirus. **C** Tumor weights were measured after the tumors were harvested from indicated groups. Data are presented as the mean ± SD from three independent experiments. *P < 0.05, **P < 0.01.

Our above data had demonstrated that ZFPM2-AS1 deficiency could suppress the cellular growth of HCC cells and promote cell apoptosis in vitro. Hence, we next sought to investigate the roles of ZFPM2-AS1 in modulating HCC tumorigenesis in vivo. First, we separately infected HepG2 cells with ZFPM2-AS1 shRNA lentivirus (including sh-ZFPM2-AS1-1 and sh-ZFPM2-AS1-2) and sh-NC lentivirus. Subsequently, the cells were collected and transplanted into nude mice. After 4 weeks, the tumor-bearing mice were killed and the subcutaneous tumors were harvested. The results of tumor growth curves confirmed that ZFPM2-AS1-1 depletion markedly reduced tumor volumes (Fig. 4A). The tumors in the sh-NC group were remarkably larger than ZFPM2-AS1 knockdown groups (Fig. 4B). In addition, repressing ZFPM2-AS1 expression resulted in significantly decreased tumor weights (Fig. 4C). Therefore, these data indicated that ZFPM2-AS1 depletion inhibited the HCC tumor growth in vivo.

### ZFPM2-AS1 deficiency attenuated the metastatic potentials of HCC cells

**Fig. 5:**
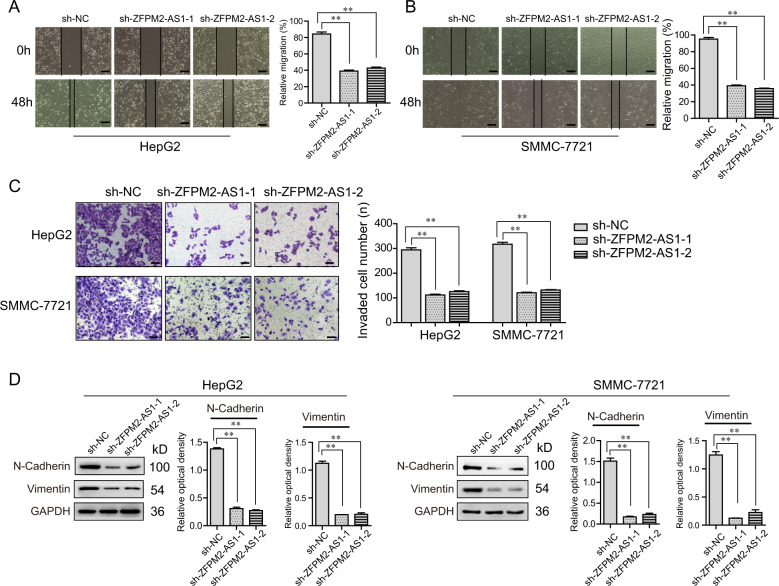
ZFPM2-AS1 promoted cell metastatic properties by affecting EMT in HCC cells. **A**, **B** The migration abilities were evaluated in HCC cells infected with sh-ZFPM2-AS1-1 or sh-ZFPM2-AS1-2 lentivirus, by wound-healing assays. Scale bar = 50 μm. **C** Transwell assays detected the invasion capacities of HCC cells after ZFPM2-AS1-2 depletion. Scale bar = 50 μm. **D** Western blot examined the protein levels of N-cadherin and vimentin in HCC cells. Data are presented as the mean ± SD from three independent experiments. *P < 0.05, **P < 0.01.

We next attempted to explore the influences of ZFPM2-AS1 on HCC metastasis. HepG2 and SMMC-7721 cells were, respectively, treated with ZFPM2-AS1 shRNAs and wound-healing assays were firstly conducted. The data indicated that ZFPM2-AS1 depletion notably reduced wound closures of HCC cells when compared with the control cells (Fig. 5A and B). Moreover, the results acquired from transwell analyses certified that suppression of ZFPM2-AS1 resulted in markedly decreased invasive cell numbers (Fig. 5C). In addition, the molecular mechanisms by which ZFPM2-AS1 silence depressed the metastatic potentials were further investigated. Epithelial to mesenchymal transition (EMT) is well known to be critical for tumor cell metastasis. Therefore, we next applied western blot to assess the protein levels of EMT relevant molecules in HCC cells after ZFPM2-AS1 was knocked down. The results clarified that ZFPM2-AS1 deficiency obviously reduced the protein levels of N-cadherin and vimentin in HCC cells (Fig. 5D). In summary, these data indicated that depletion of ZFPM2-AS1 could suppress the metastatic potentials of HCC cells via inhibiting the expression of EMT relevant molecules.

### Identification of miR‑653 as a direct target of ZFPM2-AS1 in HCC cells

**Fig. 6:**
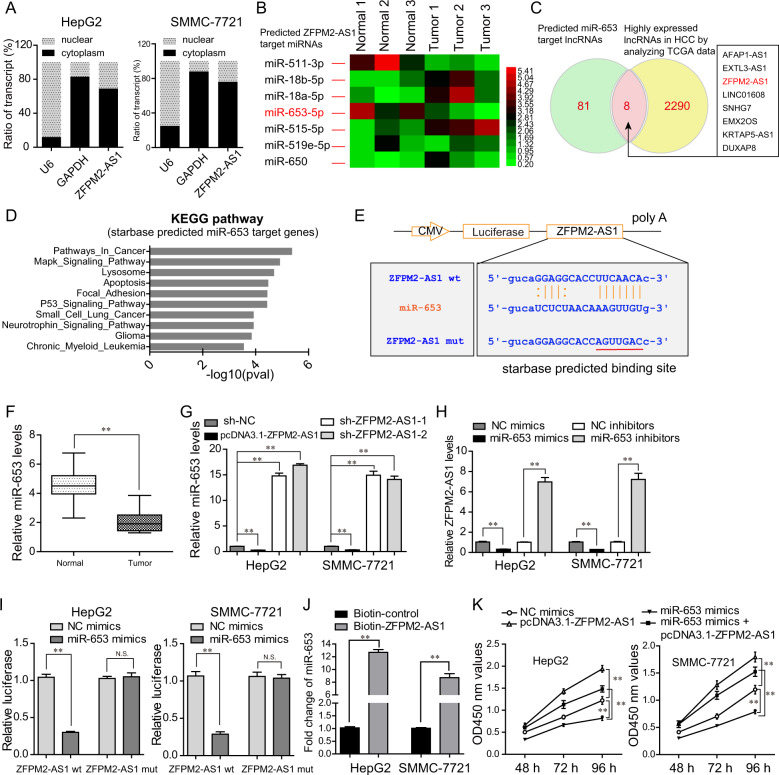
ZFPM2-AS1 acted as a sponge of miR-653 in HCC cells. **A** Subcellular location detection for ZFPM2-AS1 in HepG2 and SMMC-7721 cells. **B** Starbase algorithm predicted the target miRNAs of ZFPM2-AS1, and qPCR analyses were performed to evaluate the levels of these predicted miRNAs. The results were presented using heatmap form. **C** Venn diagram showed the intersection of predicted miR-653 target lncRNAs and highly expressed lncRNAs in HCC tumor samples. **D** Starbase algorithm analyzed the KEGG pathway of predicted miR-653 target genes. **E** The binding sites of miR-653 on ZFPM2-AS1. **F** Real-time PCR detected the relative miR-653 levels in 127 HCC samples. **G** MiR-653 levels in HCC cells were assessed by qPCR analyses when their ZFPM2-AS1 was overexpressed or knocked down. **H** ZFPM2-AS1 levels in HCC cells were assessed by qPCR analyses when their miR-653 was overexpressed or knocked down. **I** Luciferase reporter assays. **J** RNA-pulldown assays. **K** CCK-8 assays detected the cellular growth of HCC cells after various treatment. Data are presented as the mean ± SD from three independent experiments. *P < 0.05, **P < 0.01.

To further discover the potential molecular mechanisms by which ZFPM2-AS1 was able to impact the malignancy behaviors of HCC cells, we next attempted to find the downstream targets of ZFPM2-AS1. Subcellular fractionation assays revealed that ZFPM2-AS1 was mainly expressed in cytoplasm of HCC cells (Fig. 6A). Therefore, ZFPM2-AS1 might exert its oncogenic functions by acting as a miRNA sponge. Consequently, we applied “starbase” program and found that, among these predicted miRNAs, miR-511 and miR-653 were found to be downregulated in HCC tumor specimens (Fig. 6B). Considering that the functions of miR-511 in HCC had been investigated, we thereby sought to study whether miR-653, a tumor suppresser, might be a novel target of ZFPM2-AS1. Interestingly, ZFPM2-AS1 was one of the common lncRNAs included in the intersection of predicted miR-653 target lncRNAs (analyzed by “starbase” program) and highly expressed lncRNAs in HCC (Fig. 6C). Besides, KEGG pathway analyses (by “starbase” algorithm) for the predicted miR-653 target genes indicated that their targets were relevant with pathway in cancer, apoptosis, and diverse cancer types (Fig. 6D). Therefore, we next sought to clarify whether miR-653 was a direct target of ZFPM2-AS1 in HCC cells. The predicted binding site between miR-653 and ZFPM2-AS1 was presented in Fig. 6E. The qPCR assays suggested that miR-653 levels were notably downregulated in 127 HCC tumor specimens when compared with adjacent normal tissues (Fig. 6F). Moreover, forced expression of ZFPM2-AS1 led to a remarkable decrease of miR-653 expression, whereas ZFPM2-AS1 depletion markedly accelerated miR-653 expression in HCC cells (Fig. 6G). Likewise, the expression of ZFPM2-AS1 were impeded in HCC cells after transfection with miR-653 mimics, while silencing miR-653 expression could notably increase the levels of ZFPM2-AS1 (Fig. 6H). These data indicated that miR-653 expression was negatively correlated with ZFPM2-AS1 expression in HCC cells. To solidly verify that miR-653 was a target of ZFPM2-AS1, we next performed luciferase reporter assays, and the results demonstrated that miR-653 mimics dramatically depressed the luciferase activities in HCC cells when co-transfected with luciferase reporter vectors containing ZFPM2-AS1 wt but not ZFPM2-AS1 mut sequences (Fig. 6I). Furthermore, RNA-pulldown assays directly proved that ZFPM2-AS1 was capable to interacted with miR-653 in HCC cells (Fig. 6J). In addition, we next conducted CCK-8 assays to evaluate the cellular growth of HCC cells after various treatment. The data demonstrated verified that ZFPM2-AS1 was able to increase cell proliferation, whereas miR-653 could significantly suppress the growth of HCC cells. However, the inhibitory effects of miR-653 were dramatically reversed by enhancing expression of ZFPM2-AS1 in HCC cells (Fig. 6K). Collectively, the above data proved that miR‑653 was a direct target of ZFPM2-AS1 in HCC cells.

### GOLM1 served as a direct target of miR-653 in HCC cells

**Fig. 7:**
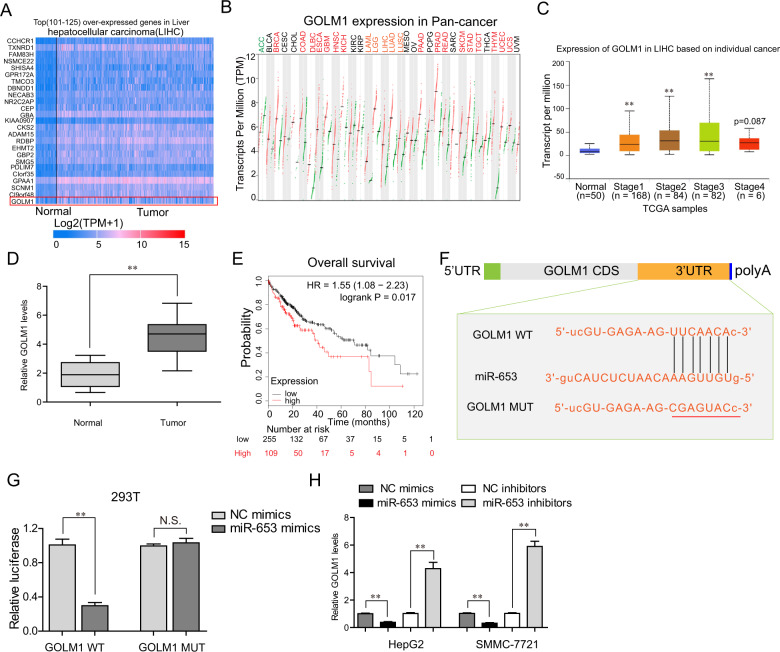
GOLM1 was a direct target of miR-653 in HCC cells. **A** UALCAN algorithm analyzed partial highly expressed lncRNAs in HCC tumor samples. GOLM1 heatmap was labeled with a red frame. **B** GEPIA algorithm analyzed GOLM1 expression across diverse cancer types. **C** UALCAN algorithm analyzed GOLM1 expression in HCC tumor specimens of patients from stage I to stage III. **D** Real-time PCR detected GOLM1 levels in 127 HCC samples. **E** Kaplan–Meier Plotter algorithm analyzed the overall survival of HCC patients with high or low GOLM1 expression. **F** Predicted binding site between miR-653 and 3’UTR of GOLM1 mRNA using strabase algorithm. **G** Luciferase reporter assays. **H** GOLM1 levels in HCC cells were assessed by qPCR analyses when their miR-653 was overexpressed or knocked down. Data are presented as the mean ± SD from three independent experiments. *P < 0.05, **P < 0.01.

Given that miRNAs exerted their functions via directly interacting with 3′-UTR of target mRNAs, we next sought to discover the downstream target genes of miR-653 in HCC. First, we profiled the differentially expressed genes in HCC using TCGA data by “UALCAN” algorithm, and found that, GOLM1, a previously reported oncogene, was highly expressed in HCC tumor samples and upregulated in most cancer types (Fig. 7A and B). Besides, bioinformatics analyses using “UALCAN” algorithm revealed that GOLM1 was remarkably elevated in HCC tumor specimens of patients from stage I to stage III (Fig. 7C). In addition, qPCR assays also validated that GOLM1 was upregulated in HCC tumor samples of 127 patients (Fig. 7D). What’s more, the “Kaplan–Meier Plotter” (http://kmplot.com/analysis/) analyses using TCGA data revealed that HCC patients with high GOLM1 levels had notably lower overall survivals (Fig. 7E). As expected, “starbase” algorithm predicted that there was a binding site between miR-653 and the 3′-UTR of GOLM1 mRNA (Fig. 7F). Therefore, we next carried out luciferase reporter assays to certify whether GOLM1 was the direct target of miR-653. The data confirmed that co-transfection with miR-653 mimics and GOLM1 WT reporter vectors notably reduced the luciferase activities in HCC cells, whereas there was no influence on the luciferase activities in cells when co-transfected with miR-653 mimics and GOLM1 MUT reporter vectors (Fig. 7G). Similarly, enhancing miR-653 expression could suppress GOLM1 levels, whereas silencing miR-653 expression was capable to promote GOLM1 levels (Fig. 7H). All the above data demonstrated that GOLM1 was a direct downstream target of miR-653 in HCC cells.

### ZFPM2-AS1 modulated GOLM1 expression via miR-653 and affected HCC cell behaviors through miR-653/GOLM1 axis

**Fig. 8:**
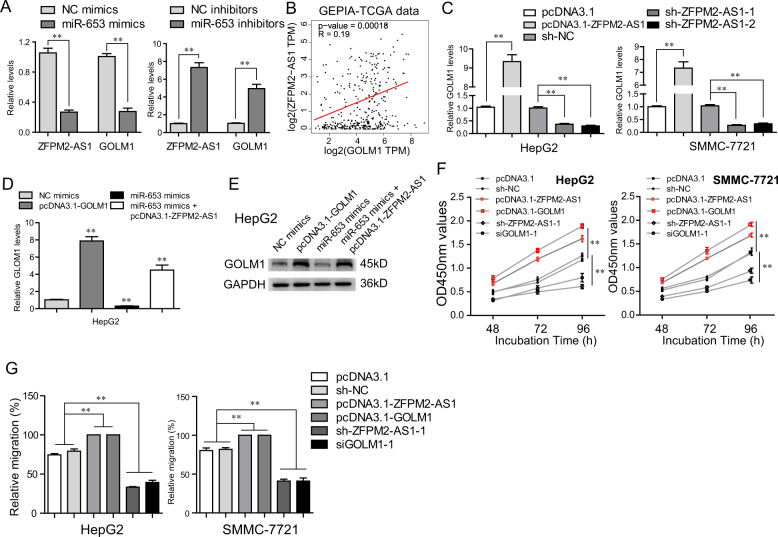
ZFPM2-AS1 modulated HCC cell malignancies via miR-653/GOLM1 axis in HCC cells. **A** ZFPM2-AS1 and GOLM1 expressing levels in HCC cells were assessed by qPCR analyses when their miR-653 was overexpressed or knocked down. **B** GEPIA algorithm analyzed the expressing correlation between ZFPM2-AS1 and GOLM1 in HCC using TCGA data. **C** GOLM1 levels in HCC cells were examined by qPCR analyses when their ZFPM2-AS1 was overexpressed or knocked down, and vice versa. **D**, **E** RT-PCR and western blot analyses measured GOLM1 protein levels in HepG2 cells. **F** CCK-8 examined the HCC cellular proliferation under various treatment. **G** Wound-healing assays detected the changes of migration capacities of HCC cells after various treatment. Data are presented as the mean ± SD from three independent experiments. *P < 0.05, **P < 0.01.

Next, we continued to study the expressing relationships among ZFPM2-AS1, miR-653, and GOLM1 in HCC cells. Real-time PCR detection validated that both the expressions of ZFPM2-AS1 and GOLM1 in HepG2 cells were repressed by miR-653 overexpression, whereas transfection with miR-653 inhibitors markedly elevated ZFPM2-AS1 and GOLM1 (Fig. 8A). Subsequently, we applied “GEPIA” algorithm (http://gepia.cancer-pku.cn/) to analyze TCGA data and found that ZFPM2-AS1 expression was positively correlated with GOLM1 levels in HCC tumor samples (Fig. 8B). The expressing relationships between ZFPM2-AS1 and GOLM1 were also demonstrated by qPCR analyses, that overexpressing ZFPM2-AS1 enhanced GOLM1 expression and silencing ZFPM2-AS1 levels markedly reduced GOLM1 expression (Fig. 8C). In addition, RT-CPR and western blot assays also revealed that ZFPM2-AS1 could abrogate the inhibitory effects of miR-653 on GOLM1 expression (Fig. 8D and E). Therefore, the above data further proved the regulatory relationships between ZFPM2-AS1 and miR-653/GOLM1 axis in HCC cells. We next sought to investigate the regulatory relationships in cellular growth and mobility between ZFPM2-AS1 and GOLM1. CCK-8 assays presented that enhancing ZFPM2-AS1 or GOLM1 could elevate the HCC cell proliferative rates, and knockdown of ZFPM2-AS1 or GOLM1 was able to attenuate the cell proliferation, which indicated that ZFPM2-AS1 and GOLM1 had similar regulatory influences on HCC cell proliferation (Fig. 8F). Similar results were also observed from wound-healing assays, that overexpression of both ZFPM2-AS1 and GOLM1 could enhancing cell wound closures (Fig. 8G). Overall, these data validated that ZFPM2-AS1 modulated HCC malignancy behaviors via miR-653/GOLM1 axis.

## Discussion

HCC has become a serious health problem with high mortality and prevalence rates in China^17^. In recent years, with the development of targeted therapies and personalized therapies, the improvement of long-term survival of HCC patients may have a breakthrough^18,19^. Sensitive diagnostic and prognostic biomarkers play a very imperative role in guiding these novel methods. Recently, lncRNAs were reported to have enormous potential as novel biomarkers^20^. In this study, we identified a novel HCC-related lncRNA, ZFPM2-AS1, which was one of the most upregulated lncRNAs in HCC by analyzing the TCGA data sets. Then, using RT-PCR, we further confirmed ZFPM2-AS1 levels were increased in HCC tissues and cell lines. Clinical investigations revealed that high expressions of ZFPM2-AS1 was associated with vein invasion, TNM stage and shorter overall survival. Importantly, the results of multivariate assays confirmed ZFPM2-AS1 as an independent poor prognostic factor for HCC patients, which highlighted its important values in clinical application.

Recently, dysregulation of ZFPM2-AS1 was also reported in several tumors, such as gastric cancer and lung cancer^14,21^. In this study, we further provided evidence that ZFPM2-AS1 was overexpressed in HCC. However, the potential mechanism remained unknown. Previous studies have suggested that lncRNAs can be activated by their upstream transcription factors. For instance, upregulation of lncRNA SPRY4-IT1 was induced by SP1^22^. Overexpression of lncRNA RAD51-AS1 was induced by E2F1^23^. To explore the mechanism involved in the overexpression of ZFPM2-AS1 in HCC, we upregulated the levels of those transcription factors to examine their influence on the levels of ZFPM2-AS1, finding that overexpression of STAT1 distinctly promoted the expressions of ZFPM2-AS1. Previously, STAT1 had been confirmed to serve as a tumor promoter and modulate the expression of several lncRNAs in various tumors^24–26^. In addition, our group performed ChIP and luciferase reporter assays, which also suggested that STAT1 could interact with the promoter of ZFPM2-AS1. Our findings indicated that STAT1 activated ZFPM2-AS1 translational expressions to realize modulation of ZFPM2-AS1 levels in HCC.

More and more evidences have confirmed that lncRNAs acted as tumor suppressors or oncogenes in tumor cells abilities. Several important lncRANs have been functionally identified, such as lncRNA MALAT1, lncRNA ANRIL, and lncRNA CASC2^27–29^. Recently, Kong et al. first, reported the oncogenic roles of ZFPM2-AS1 in gastric cancer cells^14^. To further explore the specific biological functions of ZFPM2-AS1 in HCC cells, our group decreased the expressions of ZFPM2-AS1 in HepG2 and SMMC-7721 cells and further performed a series of functional assays. We found that knockdown of ZFPM2-AS1 distinctly suppressed cells proliferation, migration, and invasion, and promoted apoptosis in vitro. In addition, the results of in vivo assays confirmed the tumor-promotive roles of ZFPM2-AS1 in HCC growth. Thus, our findings, together with previous study reporting the roles of ZFPM2-AS1 in gastric cancer, suggested ZFPM2-AS1 as a common oncogene in tumors. However, more functional assays were needed to be further confirm the roles of ZFPM2-AS1 in other tumors.

Various studies have reported that lncRNAs and miRNAs could form controlling networks for the display of their regulatory roles^30^. This new mechanism, also known as ceRNA, has emerged as vital regulator involved in epigenetic modifications, resulting in alternation of tumor-related genes via sponging miRNAs^31^. Thus, our group hypothesized that ZFPM2-AS1 could serve as a ceRNA to be involved in the progression of HCC. Using Bioinformatics tools (StarBase), we discovered seven predicted ZFPM2-AS1 target miRNAs. These miRNAs have been reported to display functional effects in cancer pathophysiology^32,33^. Of note, we further observed that miR-653 was lowly expressed in three HCC tissues by analyzing microarray data. In addition, the results of KEGG assays revealed that the targeted genes of miR-653 were positively related with the activity of several tumor-related pathways. On the other hand, overexpression of miR-653 reduced expressions of ZFPM2-AS1, whereas inhibition of miR-653 upregulated ZFPM2-AS1 expressions. More importantly, the direct binding associations between ZFPM2-AS1 and miR-653 were demonstrated by a luciferase activity assay. Overall, our findings revealed that ZFPM2-AS1 may displayed its functional significance in HCC cells by acting as a ceRNA to modulate miR-653.

As a resident cis-Golgi membrane protein, Golgi membrane protein 1 (GOLM1) has a single N-terminal transmembrane domain and coiled-coil domain^34^. In recent years, the possible effects of GOLM1 involved in the potential regulation of tumor progression attracted growing attention^35,36^. The dysregulation of GOLM1 and its oncogenic roles in several tumors have been demonstrated^37,38^. Previous, the frequent upregulation of GOLM1 in HCC patients and its prognostic values acting as a novel prognostic biomarker were also confirmed^39^. However, the molecular mechanism by which regulated thee abnormally expressed of GOLM1 in HCC remains largely unclears. In this study, GOLM1 was predicted to be a potential target of miR-653. The results of luciferase activity assays confirmed that miR-653 directly targets GOLM1 in HCC cells. In addition, we found that inhibition of miR-653 expression distinctly decreased the expressions of GOLM1, whereas upregulation of miR-653 had the opposite effects. Finally, we also provided evidence that ZFPM2-AS1 expression was positively related to GOLM1 expression and ZFPM2-AS1 could influence GOLM1 expression as ceRNAs. Functional investigations also confirmed the potential associations in the three factors.

## Conclusions

We showed that STAT1-mediated upregulation of ZFPM2-AS1 promoted HCC cell growth and metastasis through the miR-653/GOLM1 axis. The potential prognostic value of ZFPM2-AS1 was also confirmed in our clinical assays. These findings suggested that ZFPM2-AS1 may serve as a potential therapeutic target and a prognostic biomarker in HCC.

## Materials and methods

### Clinical samples’ collection

In all, 127 tumor specimens and paired adjacent normal tissues from HCC patients, who underwent surgery resection at Heilongjiang University of Chinese Medicine, were collected for this study from April 2009 to September 2012. Prior to surgery, none of the patients had received radio- or chemo- therapy. The written informed consents were obtained from all patients, and the study was approved by Ethics Committee of Heilongjiang University of Chinese Medicine. The clinical features for patients were shown in Table 1.

### Cell transfection

HCC Cells (SMMC-7721, Hep3B, HCCLM3, HepG2, MHCC97H, Huh7, QSG-7701), and LO2 cells (as control cells) were bought from Bena Culture Collection (Kunshan, Jiangsu, China). They were cultured by RPMI-1640 media (with 10% fetal bovine serum). The cell culture condition was: 37 ˚C, 5% CO_2_. The siRNAs (siRNA-control, siTNA-STAT1, siGOLM1-1, siGOLM1-2), miR-653 mimics and inhibitors were purchased from JiMa Biological corporation (Suzhou, Jiangsu, China). The overexpressing plasmids, including pcDNA3.1-STAT1, pcDNA3.1-ZFPM2-AS1, and pcDNA3.1- GOLM1, were Genetong Biological corporation (Xiamen, Fujian, China). The cell transfection was conducted using Lipofectamine 2000 reagent kits (GuangHua Biotech, Changsha, Hunan, China) in accordance with the kits’ protocols.

### Bioinformatics analyses

The HCC-related high‐throughput RNA-seq data were downloaded from The Cancer Genome Atlas (TCGA: www.tcga.org), and analyzed by R packages. The heatmap and volcano map of differentially expressed lncRNAs in HCC was also generated by R packages. JASPAR algorithm (http://jaspar.genereg.net/) was utilized to predict the binding sites between STAT1 and ZFPM2-AS1 promoter regions. The target sites between ZFPM2-AS1, GOLM1, and miR-653 were predicted by starbase algorithm (http://starbase.sysu.edu.cn/). The ZFPM2-AS1 relevant overall survival and disease-free survival of HCC patients were predicted by GEPIA algorithm (http://gepia.cancer-pku.cn/). The GOLM1-related overall survival of HCC patients were generated by Kaplan–Meier Plotter algorithm (http://kmplot.com/analysis/).

### Lentivirus production and infection

ZFPM2-AS1 shRNA sequences were, respectively, cloned into PLVX-shRNA vectors (sh-ZFPM2-AS1-1, sh-ZFPM2-AS1-2) by Genetong Biological corporation (Xiamen, Fujian, China). Then, the sh-ZFPM2-AS1-1, sh-ZFPM2-AS1-2, or control lentivirus vector was separately co-transfected with pVSVG, pRRE, and pREV plasmids into 293 T cells (70% cell confluent) using Lipofectamine 2000 reagents. The recombinant lentivirus was collected 60 hours later and filtered by the use of 0.48 μm filters. Thereafter, they were utilized for infecting cells. In short, HepG2 or SMMC-7721 cells were placed into 12-well plates. When the cell confluence reached 60–70%, 50 μl (per well) indicated lentivirus solution was added into the cells. Forty-eight hours later, the media were changed using fresh media, and the ZFPM2-AS1 shRNA lentivirus infected-cells were applied for experiments. The infection efficiency was evaluated by qPCR analyses.

### Western blot

Total proteins from ZFPM2-AS1 shRNA lentivirus infected HCC cells were extracted using cell lysis buffer (BioFeng, Changsha, Hunan, China). After the protein concentrations were determined by BCA kits (TianLi Biotech, Ningbo, Zhejiang, China), 35 micrograms of proteins were separated on sodium dodecyl sulfate polyacrylamide gel electrophoresis (8–12%), followed by transferring on to polyvinylidene difluoride membranes. Membranes were then blocked with 5% bovine serum albumin and subsequently immunoblotted at 4 °C for 10–12 h using primary antibodies: N-cadherin antibody (1:800; CST, Danvers, MA, USA); vimentin antibody (1:800; BOSTER, Wuhan, Hubei, China); GOLM1 antibody (1:1000; Abcam, Cambridge, UK); glyceraldehyde 3-phosphate dehydrogenase (abbreviated GAPDH) antibody (1:5000; PTG, Wuhan, Hubei, China). After incubating with secondary antibodies (2 h), the proteins were detected with ECL-Plus kits (Anhe Biotech, Qingdao, Jinan, China).

### Real-time PCR

RNAs were isolated by Invitrogen TRIzol reagents (Tianwei Biotech, Changsha, Hunan, China). After the concentrations of the RNAs were determined, 1 μg total RNAs were reversely transcribed into cDNA by the use of cDNA synthesis kits (DongFu Biotech, Hangzhou, Zhejiang, China). Then, qPCR detection for lncRNAs and mRNAs were conducted using TransGen SYBR Green qPCR kits (JinKaiGene, Chengdu, Sichuan, China). Results were normalized to GAPDH. The qPCR reaction conditions were: 95 °C for 5 min; 40 cycles (95 °C for 10 s, 60 °C for 15 s); 72 °C for 20 s. The relative levels of miRNAs were assayed in triplicate by the use of TransGen two-step miRNA qPCR kits (JinKaiGene, Chengdu, Sichuan, China), and U6 was used as an internal control. The qPCR results were analyzed by the 2^−ΔΔCt^ method. The primers for lncRNAs, mRNAs, and miRNAs were shown in Table 3.

**Table 3:** The primer sequences included in this study.

Name	primer sequences (5′–3′)
ZFPM2-AS1: forward	CAATGGGACTAAGCCAGGCA
ZFPM2-AS1: reverse	GGGCTCCACCAACAACCATA
STAT1: forward	CAGCTTGACTCAAAATTCCTGGA
STAT1: reverse	TGAAGATTACGCTTGCTTTTCCT
miR-653: forward	GTGTTGAAACAATCT
miR-653: reverse	GTGCAGGGTCCGAGGT
GOLM1: forward	AGAGCGTCAACAAGCTGTACC
GOLM1: reverse	CAGCCTGCCGTAATTCCTCTG
GAPDH: forward	GGAGCGAGATCCCTCCAAAAT
GAPDH: reverse	GGCTGTTGTCATACTTCTCATGG
U6: forward	CTCGCTTCGGCAGCACA
U6: reverse	AACGCTTCACGAATTTGCGT

### Cell proliferation evaluation

Cell proliferation was assessed by Dojindo CCK-8 analyses kits (DunTong Biotech, Xiamen, Fujian, China). In short, cells were harvested 48 hours after infection by ZFPM2-AS1 shRNA or control lentivirus, and placed into 96-well plates (5 × 10^3^ cells/well). Following 12 h incubation at 37 °C with 5% CO_2_, CCK-8 reagents (15 μl/well) were put into the plates. After incubation for an additional 1.5 h, the results were quantitated using a microplate reader at the wavelength of 450 nm.

### Clonogenic assays

Cells after infection with ZFPM2-AS1 shRNA or control lentivirus were collected, and, respectively, placed in six-well plates (500 cells/well). The cells were cultured in media with 10% serum in an incubator (37 °C, 5% CO_2_). The media was changed every 2 days. After culturing for two to three weeks, the colonies were treated with 95% ethanol and 0.1% crystal violet. After rinsing using PBS, the colonies were taken pictures by a microscope.

### EdU analyses

The cell proliferation was also determined by Beyotime EdU (5-Ethynyl-2’-deoxyuridine) kits (Gushang Biotech, Shenyang, Liaoning, China). The cells after infection with ZFPM2-AS1 shRNA or control lentivirus were collected and re-plated in 48-well plates. After attachment, EdU reagents (50 μl/well) were added into the cells, followed by incubation at 37 °C with 5% CO_2_ for 1.5 h. After DAPI staining and 4% paraformaldehyde fixation, the cells were then visualized under fluorescence microscopy. EdU-stained cells with green fluorescence and DAPI-stained cells (with blue fluorescence were observed.

### Flow cytometry determination

Flow cytometric analyses were carried out to detect the cell cycle and percentages of apoptotic cells. In short, the HCC cells after infection with ZFPM2-AS1 shRNA or control lentivirus were harvested in centrifuge tubes. After ice-cold PBS washing, the cells were, respectively, used for cell cycle and apoptosis analyses. For cell cycle determination, the cells were fixed at −20 °C for 12 h with ice-cold 70% ethanol. Then, the cells were treated with PI (50 μg/ml) and RNAse A (100 μg/ml) for 25 min. Then, a flow cytometer was utilized for cell cycle analysis. For cell apoptosis analyses, the cells were stained using 5 μl Annexin V/FITC reagents and 5 μl PI reagents for 25 min in the light-proof condition. After PBS washing, the percentages of apoptotic cells were analyzed with a flow cytometer. The Beyotime cell apoptosis measurement kits were bought from Gushang Biotechnology corporation (Shenyang, Liaoning, China).

### Tumor growth assay in nude mice

Twenty-four male BALB/c nude mice (5-weeks of age) were bought from Shanghai SLAC corporation (Pudong, Shanghai, China), and randomly divided into three groups. HepG2 cells were then respectively infected with sh-ZFPM2-AS1-1, sh-ZFPM2-AS1-2 or sh-NC lentivirus. Afterwards, the treated cells (1 × 10^7^ cells/mouse) were injected subcutaneously into the right flanks of mice. Tumors were allowed to grow for 28 days (4 weeks) and the mice were killed. Tumor volumes were recorded every four days with Vernier calipers. Tumor volumes were calculated using the formula: Volumes (mm^3^) = length × width^2^ × 0.5. Animal studies were approved by the Ethics Committee of our Hospital.

### Wound‑healing assay

Cells were after infection with ZFPM2-AS1 shRNA or control lentivirus were harvested and placed in twelve-well plates. On the second day, the cells were attached and the cell confluence reached ~100%. Then, micropipette tips (200 μl) were utilized to scratch across the cells. PBS was then used to wash the cells. The images of the wound closures were taken at 0 and 48 h after the wounds were generated with a microscope.

### Transwell assay

The cellular invasion ability was determined by transwell assays using Costar transwell inserts (Yunshan Biotech, Chengdu, Sichuan, China). First, the matrigel was thawed at 4 °C and placed into the inserts (75 μl/well). Then, the transwell plates were kept at 37 °C for 1.5 h. Afterwards, the remaining liquid was removed from the inserts, followed by adding 250 μl cell suspensions (without serum; 1.5 × 10^5^ cells/well) into the upside of inserts. Subsequently, 650 μl medium (per well; with 15% serum) was added into the 24-well plates. After the cells were incubated at 37 °C with 5% CO_2_ for 24 h, the cells on the lower surface of the inserts were treated with 95% ethanol and 0.1% crystal violet. After rinsing using PBS, the invasive cells were observed and taken pictures under a microscope.

### Subcellular fractionation location

The cytoplasmic and nuclear RNAs were isolated using the Life Technologies’ PARIS kits (Hongfu Biotech, Hangzhou, Zhejiang, China). In short, HCC cells (5 × 10^6^ cells) were collected, washed, and placed in 350 μl fractionation buffer. After incubation at 4 °C for 15 min, the samples were centrifuged (500×*g*/min, 4 °C, 5 min). Then, the cytoplasmic fractions were carefully aspirated away from the nuclear pellets. The nuclear pellets were then lysed using cell disruption buffer, and 2× Lysis/Binding Solution was then applied for RNA isolation. Finally, RNAs from cytoplasmic and nuclear fractions were determined by qPCR assays as described above. U6 was used as the control of nuclear transcript, and GAPDH was used as control of cytoplasmic transcript.

### RNA-pulldown

RNA-pulldown analyses were conducted using biotin-labeled ZFPM2-AS1 (Biotin-ZFPM2-AS1) as probes and then detected miR-653 by qPCR analyses. Biotin-control was used as control biotin-labeled lncRNA. In brief, cell extracts were obtained by adding lysis buffer (650 μl) into the collected cells which were infected with ZFPM2-AS1 shRNA or control lentivirus. Subsequently, 2 μg cell lysates were separately mixed with Biotin-ZFPM2-AS1 (100 pmol) or Biotin-control (100 pmol). Then, Pierce streptavidin agarose beads (Baili Biotech, Xuhui, Shanghai, China) were washed twice and 100 μl of them were, respectively, added into the above binding reactions. After the complexes were incubated at room temperature for 45 min, the beads were collected and the binding RNAs were eluted. Finally, the precipitated miR-653 was determined by RT-qPCR detection as described above.

### ChIP assay

The Millipore Magna ChIP kits (YuSheng Biotech, Jinan, Shandong, China) were utilized for ChIP assays. Then, the cells were collected and formaldehyde was applied to cross-link DNAs and proteins for 15 min. Then, glycine buffer (10×) was placed into the cells to quench the formaldehyde. Afterwards, DNA fragments (200–400 bp) were generated by sonication. The DNA fragments were then precipitated by Abcam STAT1 antibody (QiBio, Wuhan, Hubei, China). Anti-IgG antibodies (PTG, Wuhan, Hubei, China) were applied as controls. Finally, qPCR analyses were utilized for determination of precipitated DNAs.

### Luciferase reporter assay

The luciferase activities were detected by the use of Promega Luciferase reporter assay kits (Rongke Biotech, Qingdao, Shandong, China) according to the kits’ protocols. The sequence containing predicted site#2 was cloned into pGL3 luciferase reporter vector and the plasmid was named as site#2 WT (wild-type). Its matched mutant-type (MUT) luciferase reporter vector was named as site#2 MUT. Correspondingly, ZFPM2-AS1 wt luciferase reporter vector contained wild-type predicted miR-653 binding sites in ZFPM2-AS1, and ZFPM2-AS1 mut luciferase reporter vector contained the matched mutant binding sequences. In addition, the 3′-UTR of GOLM1 mRNA, or their matched mutant-type sequences were separately constructed into pGL3 luciferase reporter vectors, and named as GOLM1 WT and GOLM1 MUT, respectively. All these vectors were constructed by Keman Biotechnology corporation (Wuhan, Hubei, China).

### Statistical analyses

All statistical analyses were conducted using SPSS 20.0 (SPSS, Chicago, IL, USA). Student’s *t* test and one-way analysis of variance were respectively employed to evaluate two or multiple groups, for statistical significance. The overall survival curves were calculated with the Kaplan–Meier methods (with log-rank tests). Differences were considered statistically significant when *P* < 0.05.
